# Trends in equity in use of maternal health services in urban and rural Bangladesh

**DOI:** 10.1186/s12939-016-0311-2

**Published:** 2016-02-17

**Authors:** Nahid Kamal, Sian Curtis, Mohammad S. Hasan, Kanta Jamil

**Affiliations:** MEASURE Evaluation, University of North Carolina at Chapel Hill, Chapel Hill, USA; Seconded to International Center for Diarrheal Disease Research (icddr,b), Dhaka, Bangladesh; Department of Maternal and Child Health, Gillings School of Global Public Health, University of North Carolina at Chapel Hill, Chapel Hill, USA; Department of Population Sciences, University of Dhaka, Dhaka, Bangladesh; Office of Population, Health, Nutrition and Education, USAID, Dhaka, Bangladesh

**Keywords:** Equity, Maternal healthcare use, Urbanization, Bangladesh

## Abstract

**Background:**

Maternal healthcare utilization is a major determinant of maternal mortality. Bangladesh is experiencing a rapid pace of urbanization with all future growth in population expected to be in urban areas. Health care infrastructure is different in urban and rural areas thus warranting an examination of equity in use rates of maternal healthcare. This paper addresses whether the urban–rural and rich-poor gaps in use of selected maternal healthcare indicators have narrowed or widened over the last decade. The paper also explores changes in the service provider environment in urban and rural domains.

**Methods:**

The 2001 and 2010 Bangladesh Maternal Mortality and Health Care Survey data were used to examine trends in use of antenatal care from medically trained providers and in deliveries taking place at health facilities. Separate wealth quintiles were constructed for urban and rural areas. The concentration index was calculated for urban and rural areas to measure equity in distribution of antenatal care (ANC) and facility deliveries across wealth quintiles in urban and rural domains.

**Results:**

The gap in use of ANC provided by medically trained personnel narrowed in urban and rural areas between 2001 and 2010 while that in facility deliveries widened. The difference in use of ANC by the rich and the poor was not as pronounced as that in utilization of facilities for deliveries. Over the last decade, equity in utilization of health facilities for deliveries has improved at a faster rate in urban areas. Private sector has surpassed the public sector and appears to be the dominant provider of maternal healthcare in both domains with the share of NGOs increasing in urban areas.

**Conclusions:**

The faster pace of improvement in equity in maternal healthcare utilization in urban areas is reflective of the changing service environment in urban and rural areas, among other factors.

## Background

Bangladesh has experienced a rapid decline in maternal mortality with the maternal mortality ratio (MMR)^1^ declining from 322 (95 % CI: 259–391) to 194 (95 % CI: 149–238) deaths per 100,000 live-births between the two Bangladesh Maternal Mortality and Health Care Surveys conducted in 2001 and 2010. At this rate of decline, the country is expected to achieve Millennium Development Goal (MDG) 5 of reducing MMR to 143 deaths per 100,000 live births by 2015. This impressive improvement in maternal mortality has been largely facilitated by a government drive, with support from donor partners, to scale up the national strategy for maternal health. The strengthened efforts included upgrading of existing health facilities to provide Emergency Obstetric Care (EmOc) services since the mid 1990s and on early detection and timely referral of maternal complications [1]. The government had also revamped the Community Skilled Birth Attendants program in 2003 in order to meet the MDG target of having 50 % of all births delivered by skilled birth attendants (SBA) by 2015. Additionally, the maternal health voucher scheme, demand side financing (DSF), aimed to increase use of maternal health services among the poor, was introduced in 2007 and currently covers 53 out of a total of 490 sub-districts [2]. Maternal healthcare utilization is one of the major determinants of maternal mortality [3]. Yet at the national level, the current rate of maternal healthcare utilization is rather low – according to the 2014 Bangladesh Demographic and Health Survey (BDHS), 74 % among those who had given birth during the 3 years preceding the survey had received antenatal care (ANC) from a medically trained provider, 31 % received the recommended four or more ANC visits, 36 % of all deliveries took place at health facilities while 42 % were assisted by a skilled birth attendant (SBA) [4]. The national target is to achieve 50 % for the indicators four or more ANC visits, and SBA by 2016.

Literature on maternal healthcare utilization in South Asia reveals some distinct patterns - female healthcare utilization is lower in rural areas, socio-economic status is a primary determinant of access to maternal healthcare, and public health interventions initially increase the rich-poor gap as they tend to reach the wealthier sections of society first [5, 6, 7]. The experience of Thailand illustrates the positive role that the public sector can play in ensuring universal coverage and in reducing inequity in access to reproductive healthcare [8]. In Cambodia, inequity in use of maternal services was reduced significantly during the 2000s through pro-poor health financing and supply side policies [9].

Equity in use of maternal healthcare services has been examined primarily in the rural context in Bangladesh. These research studies generally show a large disparity in use of maternal care between the rich and poor. Chowdury et al. [10] used Matlab Demographic Surveillance System data to assess whether an intervention designed to increase facility based delivery affected equity in access to health care. They found the gap between the rich and poor to widen after the initial introduction of the intervention (promotion of facility based care) in 1996 attributable to the fact that the poor face demand as well as supply side barriers in accessing maternal healthcare. Similarly, an evaluation of a home based SBA program in rural Bangladesh showed that economic constraints are important deterrents to healthcare use - 16 % of the poorest quintile used SBA compared with 63 % of the richest quintile [11].

Approximately 40 million are estimated to be living in urban Bangladesh out of whom over 20 % or eight million live below the poverty line of US$ 2 day (UNDP, [12]). All future growth in population in the country will be driven by urban growth and the slum population is estimated to grow to 24 million by 2030. The structure for health service provision is quite different in urban and rural areas of Bangladesh. The Ministry of Health and Family Welfare's (MOHFW) public health service delivery system where primary care services are, in principle, offered for free, is not as extensive in urban areas, especially in the City Corporations. Primary health care provision in urban Bangladesh is largely under the jurisdiction of the Ministry of Local Government, Rural Development and Cooperatives. This Ministry mobilizes and outsources the provision of health services to non-governmental organizations (NGO) projects such as NGO Health Service Delivery Project (NHSDP),^2^ Urban Primary Health Care Project,^3^MANOSHI^4^and Marie Stopes.^5^ NGOs normally charge nominal fees or subsidized rates for health service provision. Secondary and tertiary levels of MOHFW health care centers are present at Thana and District Municipalities of urban Bangladesh. The gap in primary health service provision in urban areas is increasingly being filled by the private sector which, unlike the public sector, charges fees for services. With the growth of private medical colleges since the 1990s, there has been a rapid increase in private medical practice comprising pharmacies, diagnostic centers, clinics and hospitals. The share of non-formal private providers and traditional healers has also increased [13]. The growth of the private sector has been fuelled by demand side factors, in particular, by improvements in living standards.

Urban trends in maternal health care use are of particular interest given that rapid urbanization translates into increasing numbers of female migrants coming to cities. Most migrants arrive to cities with few resources. They often settle in slums with limited access to public services thus creating pockets of extreme vulnerability in urban areas. Further, the very different nature of the health care infrastructure in urban and rural areas raises questions about the implications for equity in use of maternal health services in urban and rural domains. The objectives of this paper are to examine whether equity in maternal health service use has narrowed or widened over time, and whether there are different patterns in urban and rural areas. A second objective is to examine changes in types of provider used over the last decade in urban and rural domains.

## Methods

The Bangladesh Maternal Mortality and Health Care Survey 2010, commonly known as BMMS 2010, was a multi-partner project intended to serve the programmatic and information needs of the government of Bangladesh. In order to directly compare the results of 2010 BMMS to that of 2001 BMMS, two conditions were met: 1) the sample for 2010 was designed to be large enough to detect changes from the 2001 MMR estimate with acceptable statistical precision, and 2) similar sampling procedures were employed for the two surveys. A sample size of 175,000 households was estimated to be large enough to detect roughly a 20 % decline in the 2001 MMR estimate of 322 deaths per 100,000 live births with 95 % significance and 80 % power [14]. Maternal health care questions were asked of women who had a live birth in the preceding 3 years. For the 2010 BMMS survey, questions on maternal healthcare use were included in the long questionnaire which was administered to a sub-sample of approximately 62,000 ever-married women aged 13–49. Questions on maternal healthcare utilization were asked to over 104,000 ever-married female respondents aged 13–49 in the 2001 BMMS survey.

The 2001 BMMS used three questionnaires (household questionnaire, women's questionnaire and verbal autopsy form) whereas the 2010 BMMS employed five (household questionnaire, women’s short questionnaire, women’s long questionnaire, verbal autopsy questionnaire and community skilled birth attendants’ questionnaire). The main themes and structures for the two surveys were kept similar for general comparability. However, the 2001 and 2010 BMMS surveys were not identical in terms of contents or in the way some questions were asked. This somewhat restricted the extent to which comparative analysis could be undertaken. For example, the 2001 BMMS did not ask questions on source/type of antenatal care provider in a manner to enable analysis by public, private and NGO sectors. For SBA, the BMMS survey in 2001 asked about the provider who had assisted with the last delivery whereas the survey in 2010 had broken the question down into two parts: a) who actually performed the delivery, and b) who assisted with the delivery. It was not possible to compare trends in postnatal care as the 2001 and 2010 BMMS questionnaires defined the reference period for postnatal care differently. Given these differences between the two surveys, results presented here focus on two indicators - ANC from medically trained personnel and deliveries at health facilities.

For the purpose of this analysis, separate wealth quintiles were constructed for urban and rural areas for both 2001 and 2010 BMMS datasets by assigning different weights to the asset variables. This was done to account for the fact that living standards are different in urban and rural contexts. The wealth quintile is a measure of economic status based on an asset score of household assets, and household characteristics. The distribution of the household asset score is divided into quintiles from poorest to richest such that, 20 % of sampled households fall in each quintile. The standard method of principle component analysis was used to generate the wealth index. It is a widely used proxy for economic status in lieu of income/expenditure data (see, for example, [15]).

For each of the two indicators of maternal healthcare use that were examined in this paper, we looked at change in absolute use in urban and rural areas between 2001 and 2010 as well as change in types of providers in urban and rural domains. We calculated *p*-values to examine if these changes between 2001 and 2010 were statistically significant. To examine change in equity, we looked at three conventional indicators - change in absolute use between the richest and poorest quintiles, change in the poor-rich ratio in use of ANC and facility deliveries and finally the concentration index which takes into account all the wealth quintiles. The concentration index is a measure of the distribution of a specific health variable among different socio-economic groups. It has been used widely to measure equity of a distribution across wealth quintiles *vis a vis* indicators of health in low and middle income countries [16, 17]. It is based on a concentration curve that is obtained by plotting the cumulative proportion of the population ranked by wealth status on the x axis and the cumulative proportion of the outcome of interest in the y axis. A concentration index of 0 implies perfect equity; positive values denote skewness towards richer quintiles while negative values indicate skewness towards the poorer quintiles.

## Results

### Antenatal care from medically trained provider

**Fig. 1:**
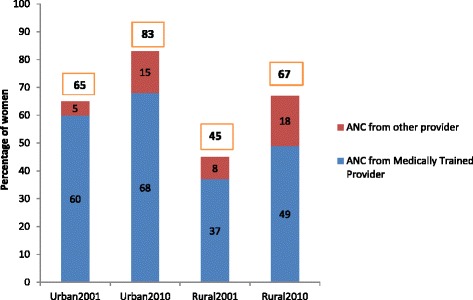
Change in Use of Antenatal Care in Urban and Rural Areas

Use of ANC provided by medically trained providers (MTP) was already quite high in urban areas in 2001 at 60 % compared with 37 % in rural areas (Fig. 1). By 2010, these urban and rural rates had increased to 68 and 49 %, respectively, with the urban–rural gap in use of ANC narrowing. The increase in use of ANC provided by MTP in urban and rural areas over this period was statistically significant at the 5 % level (Table 1). Although our focus is on ANC provided by MTP, the simultaneous increase in the level of ANC provided by non-MTP by ten percentage points in both urban and rural domains is noteworthy. In fact, in urban areas, use of ANC by MTP increased by eight whereas use in ANC provided by non-medically trained personnel increased by 10 percentage points. Our analysis further found that 36 % of pregnant women in urban versus almost 20 % in rural areas had gone for the recommended 4+ ANC visits in 2010 (data not shown).

**Table 1:** Antenatal Care. Percentage of women aged 15–49 with a live birth in the 3 years preceding survey who received at least one Antenatal Care for their most recent pregnancy, by medically trained provider and by wealth quintile, BMMS 2001 and 2010

	Urban								Rural							
Household wealth index	Medically trained providers^a^			Non-medically trained providers^b^			Number of women		Medically trained providers^a^			Non-medically trained providers^b^			Number of women	
	2001	2010	*p*-value	2001	2010	*p*-value	2001	2010	2001	2010	*p*-value	2001	2010	*p*-value	2001	2010
Poorest	31.5	46.4	0.00	7.3	20.0	0.00	1426	799	21.7	28.2	0.00	9.0	22.8		6335	2706
Poorer	45.4	61.7	0.00	7.2	16.6	0.00	1364	793	26.9	35.8	0.00	8.9	24.0	0.00	6001	2577
Middle	60.3	65.9	0.01	5.7	17.3	0.00	1304	834	32.4	46.4	0.00	8.6	19.8	0.00	5855	2565
Richer	79.8	72.2	0.00	3.7	15.8	0.00	1156	805	42.0	55.8	0.00	7.5	16.3	0.00	5758	2572
Richest	94.4	93.6	0.40	1.4	3.2	0.01	1106	762	62.8	76.5	0.00	5.2	9.1	0.00	5971	2735
Total	60.1	67.7	0.00	5.3	14.7	0.00	6356	3994	36.9	48.7	0.00	7.9	18.3	0.00	29920	13156
Concentration index	0.21	0.12	-	−0.22	−0.18	-	-	-	0.21	0.19	-	−0.10	−0.16	-	-	-

^a^Includes Qualified doctors, Nurses/midwife/paramedics/FWV, MA/SACMO

^b^Includes Community Health Workers(HA, FWA, BRAC & Other NGO Health Providers),Other

**Fig. 2:**
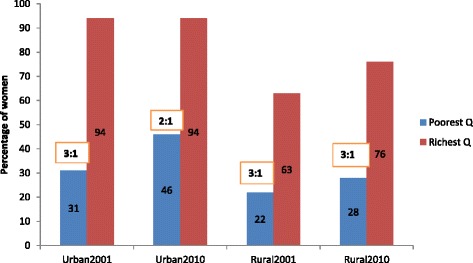
Rich-poor Gap in Antenatal Care in Urban and Rural Areas

Figure 2 presents the proportion of women from the richest and poorest quintiles that used ANC provided by MTP. In 2001, roughly three times as many pregnant women from the richest quintile as from the poorest quintile sought ANC from a MTP in urban areas, 94 and 31 %, respectively. Urban areas witnessed a 15 percentage point increase in use by the poorest quintile over the last decade thus improving the rich-poor ratio to 2:1. In rural areas, the increase in use of ANC provided by MTP among the poorest quintile was by six percentage points from 22 to 28 % while that among the richest was by 13 percentage points from 63 to 76 %. The rural rich-poor ratio in use of ANC remained unchanged at 3:1 in 2010 attributable to the relatively lower level of increase in use of ANC among the poorest group in rural Bangladesh.

**Fig. 3:**
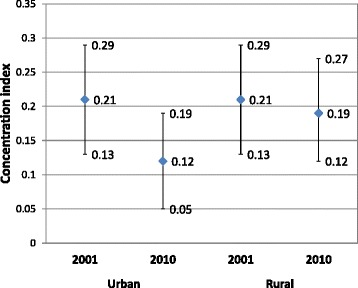
Concentration Index of Antenatal Care by Medically Trained Providers with 95 % Confidence Intervals

The concentration index is a more refined measure of health inequity which shows the distribution of use across all the five socio-economic groups or wealth quintiles (Fig. 3). An index of 0.21 (95 % CI: 0.13–0.29) was estimated for antenatal care for both urban and rural areas for 2001 suggesting a positive skewness towards the rich. By 2010, this index had declined to 0.12 (95 % CI: 0.05–0.19) in urban areas and to 0.19 (95 % CI: 0.12–0.27) in rural areas implying an improvement in equity, more so in urban areas. However, the overlap in the confidence intervals around the concentration indices of the 2001 and 2010 urban and rural point estimates indicate that the apparent improvement in equity over time was not statistically significant at the 5 % level.

**Fig. 4:**
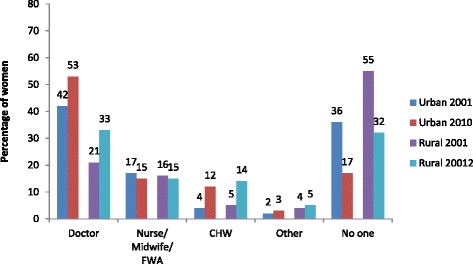
Change in Type of Provider of Antenatal Care in Urban and Rural Areas

There is a marked increase in use of medical doctors in both urban and rural areas between the two surveys (Fig. 4). The use of Community Health Workers (CHW) was also high in 2010 relative to 2001. The patterns in use of public versus private sector for ANC by wealth quintile is pretty similar in urban and rural areas, that is, there is a shift from public to private sectors with increasing wealth (Table 2). What is most different between urban and rural areas is in use of the NGO sector, which is higher in urban areas and does not vary that much by wealth status in either area.

**Table 2:** Type of provider of Antenatal Care. Percent distribution of women with a live birth in the 3 years preceding survey who received Antenatal care from a medically trained provider at least once during their most recent pregnancy by type of provider and wealth index, urban and rural areas, BMMS 2010

	Urban				Rural			
	Public	Private	NGO	Number of women	Public	Private	NGO	Number of women
	2010	2010	2010	2010	2010	2010	2010	2010
Household wealth index								
Poorest	67.7	19.3	17.2	122	68.1	26.0	7.6	1028
Poorer	64.4	18.4	20.9	182	63.8	31.7	7.5	1171
Middle	50.7	32.4	22.7	285	56.7	41.2	8.4	1475
Richer	40.3	43.1	24.6	572	46.4	51.7	10.0	1604
Richest	31.9	55.7	21.9	1543	34.5	65.6	7.9	1126
Total	39.5	46.4	22.3	2703	53.4	44.0	8.4	6405

Multiple responses possible

### Place of delivery

**Fig. 5:**
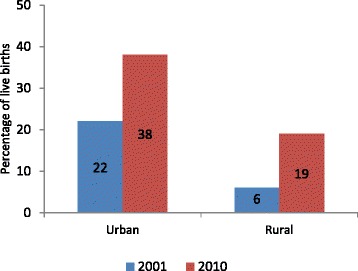
Change in Facility Deliveries in Urban and Rural Areas

In 2001, the percentage of deliveries that took place at a health facility was over four times higher (22 % versus six percent) in urban areas than in rural areas (Fig. 5). The percentage of institutional deliveries increased from roughly 22 to 38 % in urban areas and from 6 to 19 % in rural areas between the two surveys. This increase in use rates was statistically significant at the 5 % level. The urban–rural gap in absolute use of this indicator widened from 16 to 19 percentage points over the last decade when it narrowed for ANC provided by MTP, as mentioned earlier.

**Fig. 6:**
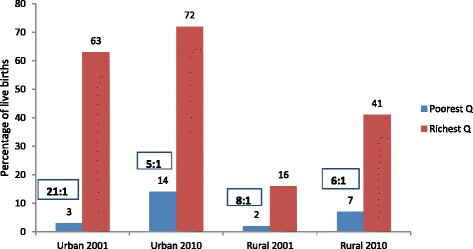
Rich-Poor gap in Facility Deliveries in Urban and Rural Areas

An examination by wealth quintile exposes stark inequality in use of facilities for deliveries by the rich and the poor in urban Bangladesh (Fig. 6). In 2001, institutional delivery was negligible among the poorest quintile – only three percent of women from the lowest quintile delivered at facilities compared with 63 % from the highest quintile. The rich-poor ratio in the prevalence of facility deliveries narrowed remarkably from 21:1 in 2001 to 5:1 in 2010 while the absolute difference between the richest and poorest quintiles also narrowed slightly from 60 to 58 percentage points. In rural areas, facility deliveries continue to be low among all quintiles. Although the rich-poor ratio in rural areas had narrowed from 8:1 to 6:1 between the two surveys, the absolute difference in use of facility deliveries by the richest and poorest had increased from 14 to 34 percentage points as relatively more of the wealthier rural residents began to use facilities for delivery care. Thus while the urban poor are increasingly availing of health facilities for deliveries, the rural poor are still lagging behind.

The concentration index for facility deliveries in urban areas was skewed towards the rich at 0.51 (95 % CI: 0.43–0.59) in 2001 but almost halved to 0.27 (95 % CI: 0.19–0.30) in 2010 confirming a significant improvement in equity in use of institutional deliveries in urban areas over the last decade. The concentration index in rural areas declined from 0.41 (95 % CI: 0.33–0.49) to 0.34 (CI 95 %: 0.29–0.38) between the two surveys. Unlike in urban areas, the improvement in equity in use of facility deliveries in rural areas was not statistically significant.

**Fig. 7:**
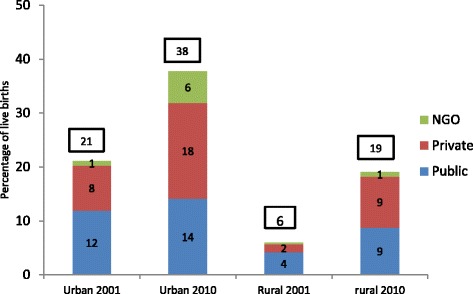
Change in Place of Delivery in Urban and Rural Areas

Finally looking at the type of facility used for delivery, the share of the private sector increased in both urban and rural areas and surpassed that of the public sector to stand as the dominant provider in 2010 (Fig. 7). Use of the NGO sector for facility deliveries has increased from 1 to 6 % in urban areas whereas it remains almost non-existent in rural areas. Among those who deliver at facilities, it is the middle three quintiles that are most likely to use NGOs - around 20 %. Utilization by wealth quintile shows that women from the poorer households opt for public facilities while those from wealthier households use private health centers (Tables 3 and 4). However share of the private sector appears to have also increased among the poorer quintiles over the last decade.^6^

**Table 3:** Type of Place of delivery (URBAN). Percent distribution of live births in the 3 years preceding survey by type of place of delivery and wealth quintile, BMMS 2001 and 2010

	Public			Private			NGO			Non-facility delivery^a^			Number of births	
	2001	2010	*p*-value	2001	2010	*p*-value	2001	2010	*p*-value	2001	2010	*p*-value	2001	2010
Household wealth index														
Poorest	2.8	9.5	0.00	0.5	3.2	0.00	0.1	1.5	0.00	96.5	85.8	0.00	1559	851
Poorer	4.9	13.7	0.00	0.9	10.8	0.00	0.1	3.6	0.00	94.1	71.9	0.00	1486	845
Middle	10.5	13.4	0.04	3.1	14.3	0.00	0.2	7.1	0.00	86.2	65.3	0.00	1403	875
Richer	20.1	13.7	0.00	9.7	20.9	0.00	1.7	7.6	0.00	68.5	57.8	0.00	1212	838
Richest	26.2	20.4	0.00	34.1	41.8	0.00	2.4	9.3	0.00	37.4	28.6	0.00	1149	795
Total	11.9	14.1	0.00	8.4	17.8	0.00	0.8	5.8	0.00	78.9	62.3	0.00	6809	4203
Concentration Index	0.40	0.12	-	0.66	0.38	-	0.56	0.27	-	−0.14	−0.16	-	-	-

^a^Includes Home, Others

**Table 4:** Type of Place of delivery (RURAL). Percent distribution of live births in the 3 years preceding survey by type of place delivery and wealth quintile, BMMS 2001 and 2010

Characteristics	Public			Private			NGO			Non-facility delivery^a^			Number of births	
	2001	2010	*p*-value	2001	2010	*p*-value	2001	2010	*p*-value	2001	2010	*p*-value	2001	2010
Household wealth index														
Poorest	1.5	3.6	0.00	0.1	2.7	0.00	0.1	0.5	0.00	98.3	93.3	0.00	6972	2931
Poorer	2.5	7.0	0.00	0.4	3.0	0.00	0.2	0.7	0.00	96.9	89.3	0.00	6585	2783
Middle	3.0	8.0	0.00	0.6	7.0	0.00	0.1	0.8	0.00	96.3	84.2	0.00	6373	2715
Richer	4.4	10.2	0.00	1.2	10.1	0.00	0.4	0.8	0.01	94.1	78.9	0.00	6227	2702
Richest	9.7	15.3	0.00	5.1	24.1	0.00	0.8	1.7	0.00	84.4	58.9	0.00	6376	2902
Total	4.2	8.8	0.00	1.5	9.4	0.00	0.3	0.9	0.00	94.1	80.9	0.00	32534	14033
Concentration index	0.35	0.25	-	0.59	0.43	-	0.4	0.23	-	−0.03	−0.08	-	-	-

^a^Includes Home, Others

## Discussion

This paper set out to examine how equity in maternal healthcare utilization has changed between 2001 and 2010 and whether the patterns are different in urban and rural areas. The main finding is that equity has improved at a faster rate in urban areas, and this appears to be at least partially reflective of the different service environments in urban and rural domains of the country. The main results and implications are discussed for each of the two indicators separately and then placed in the context of the key changes in the service environment.

Use of ANC from medically trained providers increased between 2001 and 2010 and the urban–rural gap narrowed. The gap in use between the rich and poor remained unchanged in rural areas in 2010 but improved in urban areas. Framing of questions in the 2001 and 2010 surveys was slightly different and does not allow us to directly compare changes in public, private and NGO sectors over time. Nevertheless some conclusions can still be drawn by looking at type of provider. The largest increase was in use of medical doctors for ANC in both urban and rural domains and private facilities in Bangladesh are most likely to be staffed by medical practitioners [18]. There is anecdotal evidence to suggest that women consider an ultra-sound to be an integral part of an ANC check-up. Private facilities are more likely than public or NGO ones to be equipped to provide ultra-sound services which may be contributing to the recent shift in preference for doctors when seeking antenatal care. However, the poorest three quintiles in both urban and rural Bangladesh were most likely to seek ANC services from the public sector in 2010 when they did seek care. The second largest increase was in use of CHWs, a cadre of outreach workers belonging mainly to NGOs. The threefold increase observed in use of CHWs for ANC in both urban and rural areas is suggestive of an increased use of the NGO sector. These providers have no formal medical training to provide ANC which raises potential concerns about the quality of ANC provided. The preliminary findings from the 2013 Bangladesh Urban Health Survey (BUHS) show that non-MTPs are less likely to provide urine and blood tests and an ultra-sonogram, three out of the five essential components of ANC [19].

Use of facilities for deliveries has increased by a greater margin in urban areas than in rural areas resulting in a widening of the urban–rural gap in this indicator over the last decade. In 2001, facility deliveries were almost non-existent among the poorest quintile in both urban and rural areas. The use of facilities for delivery among the urban poor increased rapidly and in 2010, the rich-poor ratio in facility delivery coverage in urban areas had narrowed considerably. In contrast, the rich-poor gap in delivery care has widened in absolute terms in rural areas as notable increases in use among wealthier quintiles have not been matched by similar gains in poorer quintiles.

The fast pace of increase in uptake of institutional deliveries among the urban poor deserves a closer look. Use-inequity is believed to be low in settings where service coverage is high [2]. Donor organizations like USAID and DFID have made provision of urban maternal care services a priority area for support in recent years. Programs like NHSDP, UPHCP and MANOSHI through their static and outreach service delivery models have targeted the urban poor. The NHSDP aims to make 20 % of all service contacts with the poor and has a provision for Health Benefit Cards which poor customers can use to receive health services at subsidized prices or for free. UPHCP operates on a similar mandate of targeting the poor. The BRAC operated MANOSHI project has had an extensive outreach arm since 2007 for identifying and tracking pregnant women among the urban poor and making referrals for the complicated cases to government health centers in addition to its network of birthing huts and BRAC static centers in slum settlements. Findings from the 2013 BUHS reveal that one in four facility births in slums take place at BRAC facilities [19].^7^ These initiatives by the NGO sector are likely to have contributed to the increase in delivery care in the NGO sector in urban areas observed in this study but potentially also indirectly to the increase in delivery care in other sectors through referral mechanisms. The overall coverage of NGOs is still low - in Dhaka slum settlements, only 20 % of the health service delivery points were found to be owned by NGO or public providers [13].

A significant part of the increase in use of delivery care in urban areas has been in use of the private sector including among the urban poor. Use of private facilities has also increased substantially among the wealthier quintiles in rural areas, contributing to widening differentials in use of delivery care there. However, the large increase in use of the private sector raises concerns over financial burden on poor families. Some NGO and private clinics are part of the Demand Side Financing program which is a maternal health voucher program initiated by the government with support from WHO to increase utilization of maternal healthcare especially by the poor. Various research studies assessing this program document its effectiveness in increasing use rate of maternal healthcare services among the poorer sections of the population [20, 11]. Nevertheless, it has been estimated that 44 % of poor households who delivered at a health facility experienced catastrophic expenditures associated with maternity care compared to three percent of poor households who delivered at home [21]. Another concern with facility-based deliveries is the potential for over-medicalization of delivery care, particularly in response to profit motivations in the private sector. Findings of the 2013 BUHS reveal that 44 % of facility deliveries in urban slum settlements and 65 % of those in non-slum areas of City Corporations were conducted through Cesarean-section when WHO guidelines advocate that the Cesarean-section rate not exceed 15 %.

The public sector remains an important source of delivery care, particularly in rural areas and among the poor. The government program had embarked upon scaling up the national strategy for maternal health primarily through upgrading the existing facilities to provide EmOc services. There is evidence to suggest that the strengthened program on maternal care, as part of Averting Maternal Deaths and Disability project did lead to an increase in use of reproductive health services in the country [1]. While overall use of both ANC and delivery care have increased, a large rich-poor gap prevails in utilization of maternal healthcare. Large scale implementation of pro-poor policies and interventions may be needed in the government program to increase use among the poor, particularly in rural areas.

One of the limitations of this study is that it does not provide an insight into why and how the poor choose the type of provider that they do. According to findings of the 2013 BUHS, the cost of a normal delivery is lowest at a NGO facility and highest at a private facility (up to five times higher) in urban areas [19] and yet the private sector has a greater share than NGOs in use of delivery care among the poorest two quintiles in both domains. This suggests that non-economic factors such as accessibility, convenience and quality play a determining role in choice of provider. Studies on health care utilization in India conclude that the poor are increasingly turning to the private sector because paid services, as opposed to free services provided in the government program, are perceived to be of better quality [22]. A second limitation is that the poorest quintile in urban areas considered in this analysis does not necessarily reflect the urban poor in slum settlements as this sub-group was not oversampled in the BMMS surveys. Finally, this study provides little information on the demand side factors that may have contributed to an overall increase in maternal healthcare utilization in the country. A number of non-health sector related factors like an increase in female education, economic prosperity and improved communication are known to have facilitated the gains in maternal mortality during the last decade [23].

## Conclusions

Although use of ANC and facility deliveries have increased substantially over the period 2001 to 2010, the coverage of these maternal health services is well below the national targets set for 2016 and there has been little change in the absolute difference in coverage between urban and rural areas. The rich-poor gap in maternal healthcare use seems to be closing at a faster rate in urban than in rural Bangladesh especially for ANC. Inequity however continues to prevail, more so in rural areas. The different service environments in urban and rural areas at least partially explain these trends but further research is needed to better understand the relative contribution of specific pro-poor policies and programs, and of larger demand side factors.

The main implication of the study is that choice of service provider could potentially facilitate or hinder equitable use of health services. Routine evaluation of the mix of public/private/NGO providers may be beneficial in making healthcare accessible to all. Equity in use of maternal healthcare in rural Bangladesh could be improved by offering a more balanced choice of service providers, rather than relying primarily on any one sector for provision of health services.

## Abbreviations

ANC: Antenatal care
BDHS: 2014 Bangladesh Demographic and Health Survey
BRAC: *formerly* Bangladesh Rural Advancement Committee
BUHS: 2013 Bangladesh Urban Health Survey
CHW: community health worker
CI: confidence intervals
DFID: UK Department for International Development
DSF: demand side financing
EmOC: emergency obstetric care
MDG: millennium development goal
MMR: maternal mortality ratio
MOHFW: Ministry of Health and Family Welfare
MTP: medically trained provider
NGO: non-government organization
NHSDP: NGO health service delivery project
SBA: skilled birth attendant
UPHCP: urban primary health care project
USAID: U.S. Agency for International Development
WHO: World Health Organization

## Footnotes

1 Maternal mortality ratio (MMR) measures the mortality risk associated with each live birth.

2 NGO Health Service Delivery Project (commonly known as Smiling Sun) is USAID'S flagship NGO healthcare delivery program that provides primarily family planning and maternal, newborn and child health services to a catchment population of roughly 20 million. With recent DFID funding, the project expects to make major expansions in the urban component of the program by the end of the current phase in 2017. Health services are provided through 392 static clinics, over 10,000 satellite sessions and over 6,000 community health workers in urban as well as rural areas. Out of the total 207 static clinics in urban areas, 44 provide comprehensive emergency and obstetric care services while another six offer basic EmOc as of 2015.

3 Urban Primary Health Care Project (UPHCP) Phases 1 (1998–2005) and 2 (2005–2011) were initially funded by ADB, SIDA, DFID and UNFPA operating in 6 City Corporations and 5 Municipalities. At least 30 % of contacts are targeted towards the poor. The project contracts out primary healthcare services to local selected NGOs just like Smiling Sun does. The current Phase 3 (2012–2017) covers around ten million in urban areas through a network of 25 Comprehensive Reproductive Health care Centers, 112 Primary Heath Care Centers and 224 satellite clinics.

4 MANOSHI is a community based program implemented by BRAC which provides a comprehensive package of essential services for maternal, newborn and child health in urban slums. The total anticipated coverage was eight million across all slums in Dhaka Metropolitan Area by 2009 and the program currently covers all nine City Corporations of the country. The model deploys two kinds of health workers in the community -*Shastho Shebika* or community workers and *Shastho Kormi*s who are equipped with mobile phone based data collection software. Medical doctors then provide feedback on the information collected by *Shastho Kormi*s on mothers and children. In addition, birthing huts were set up in slums. The system relies on quick action on maternal health emergencies. Evaluation of the initial 2007–2011 phase revealed that the percentage of institutional deliveries (including in BRAC birthing huts) increased from 15 to 59 % in project areas and from 25 to 28 % in comparison areas.

5 Marie Stopes, an affiliate of Marie Stopes International (MSI) started operating in Bangladesh in 1988. Today, MSI’s program in the country is one of the largest in the MSI global partnership with 144 clinics in addition to outreach sites and mobile services covering 62 out of 64 districts in the country. The MSI clinics provide sexual and reproductive health services to 1.4 million Bangladeshis each year. It operates 141 clinics in urban and peri-urban areas out of which 15 provide maternal services including deliveries by C-section.

6 Patterns in use levels and equity for SBA are very similar to that for facility deliveries presented in this paper.

7 Deliveries in BRAC birthing huts are not attended by a SBA, however, given that these birthing huts are not staffed by medically trained practitioners.

## References

[1] Gill Z, Ahmed JU (2004). Experience from Bangladesh: implementing emergency obstetric care as part of the reproductive health agenda. Int J Gynecology and Obstetrics.

[2] Anwar I, Nababan HY, Mostari S, Rahman A, Khan JAM (2015). Trends and Inequities in Use of Maternal Health Care Services in Bangladesh, 1991–2011. PLoS ONE.

[3] McCarthy J, Maine D (1992). A Framework for Analyzing the Determinants of Maternal Mortality. Stud Fam Plann.

[4] National Institute of Population Research and Training (NIPORT), Mitra and Associates, & ICF International (2015). Bangladesh Demographic and Health Survey 2014: Key Indicators.

[5] Liang J, Zhu J, Zeng J, Li X, Zeng W, Li Q (2011). Preventable maternal mortality: Geographical/rural–urban differences and associated factors from the population based maternal mortality surveillance system in China. BMC Public Health.

[6] Sannevig L, Trygg N, Saxena D, Mavalankar D, Thomsen S (2013). Inequity in India: the case of maternal and reproductive health. Global Health Action.

[7] Simkhada B, Teijlingen ER, Porter M, Simkhada P (2008). Factors affecting the utilization of antenatal care in developing countries: systematic review of the literature. J Adv Nurs.

[8] Kongsri S, Limwattananon S, Sirilak S, Pralongsai P, Tangcharoensathien V (2011). Equity of access to and utilization of reproductive health services in Thailand: national Reproductive Health Survey, 2006 and 2009. Reprod Health Matters.

[9] Dingle A, Powell-Jackson T, Goodman C (2013). A decade of improvements in equity of access to reproductive and maternal health services in Cambodia, 2000–2010. Int J Equity Health.

[10] Chowdury ME, Ronsman C, Killewo J, Anwar I, Gausia K, Das-Gupta S (2006). Equity in use of home-based or facility-based skilled obstetric care in rural Bangladesh: an observational study. Lancet.

[11] Anwar I, Sami M, Akhtar A, Chowdhury M, Salma U, Rahman M (2008). Inequity in maternal health care services: evidence from home based skilled-birth-attendant programmes in Bangladesh. Bulletin of World Health Organization.

[12] UNDP 2013. http://www.bd.undp.org/content/bangladesh/en/home/presscenter/articles/2013/10/03/recognizing-slums-in-bangladesh/. [Accessed 12 April 2015].

[13] Adams AM, Islam R, Ahmed T (2015). Who serves the urban poor? A geospatial and descriptive analysis of health services in slum settlements in Dhaka Bangladesh. Health Policy Plan.

[14] National Institute for Population Research and Training (NIPORT), MEASURE Evaluation, International Centre for Diarrheal Diseases Research (icddr,b) (2012). Bangladesh Maternal Mortality and Health Care Survey 2010.

[15] Filmer D, Pritchett L (2011). Estimating Wealth Effects Without Expenditure Data - or Tears. An Application to Educational Enrollments in States of India. Demography.

[16] Baqui AH, Rosecrans AM, Williams EK, Agrawal PK, Ahmed S, Darmstadt GL (2008). NGO facilitation of a government community-based maternal and neonatal health programme in rural India: improvements in equity. Health Policy Plan.

[17] Quayyum Z, Khan MN, Quayyum T, Nasreen H, Chowdhury M, Ensor T (2013). Can community level interventions have an impact on equity and utilization of maternal health care - Evidence from rural Bangladesh. Int J Equity Health.

[18] National Institute of Population Research and Training (NIPORT), Associated for Community and Population Research (ACPR), & ICF International (2015). Bangladesh Health Facility Survey 2014: Preliminary Findings.

[19] National Institute for Population Research and Training (NIPORT), International Centre for Diarrheal Disease Research (icddr,b), & MEASURE Evaluation (2015). Bangladesh Urban Health Survey 2013 Final Report.

[20] Ahmed S, Khan MM (2011). Is demand side financing equity enhancing? Lessons from a maternal health voucher scheme in Bangladesh. Soc Sci Med.

[21] Brugh K, Angeles G (2014). Analysis of Maternal Health Expenditures by Services Received, Poverty Status, and Divisions.

[22] Misra R, Chatterjee R, Rao S (2003). India Health Report.

[23] El Arifeen S, Hill K, Ahsan KZ, Jamil K, Nahar Q, Streatfield PK (2014). Maternal mortality in Bangladesh: a Countdown to 2015 country case study. Lancet.

